# Laboratory and in-situ investigations for trapping Pb and Ni with an unusual electrochemical device, the calcareous deposit in seawater

**DOI:** 10.1038/s41598-019-40307-0

**Published:** 2019-03-04

**Authors:** Charlotte Carré, Peggy Gunkel-Grillon, Arnaud Serres, Marc Jeannin, René Sabot, Thomas Quiniou

**Affiliations:** 1 Laboratoire des Sciences de l’Ingénieur pour l’Environnement LaSIE UMR-CNRS-7356 - Université de La Rochelle, La Rochelle, France; 2 Institut des Sciences Exactes et Appliquées ISEA EA-7484 - Université de la Nouvelle Calédonie, New Caledonia, France

## Abstract

In seawater, the application of a cathodic current in a metallic structure induces the formation of a calcareous deposit formed by co-precipitation of CaCO_3_ and Mg(OH)_2_ on the metal surface. A previous study proved that this electrochemical technique is convincing as a remediation tool for dissolved nickel in seawater and that it is trapped as nickel hydroxide in the deposit. Here, the precipitation of a carbonate form with lead is studied. Pb^2+^ precipitation in calcareous deposit was investigated with a galvanized steel electrode by doping artificial seawater with PbCl_2_. Results show for the first time the presence of Pb incorporated in its carbonate form in the calcareous deposit. Trapped Pb content increased with initial Pb content in seawater. Simultaneous doping with Ni and Pb revealed that Ni trapping was favoured by higher current densities while Pb trapping was favoured by lower current densities. Finally, preliminary *in situ* experiments were performed in an industrial bay and validated the incorporation in real conditions of contaminants by precipitation with the calcareous deposit The present work demonstrates that co-precipitation of contaminants under their hydroxide or carbonate form in a calcareous deposit is a promising clean-up device for remediation of contaminated seawater.

## Introduction

Although metallic heavy metals are naturally present in the environment, they can significantly affect marine ecosystems and human health at high concentrations^1,2^. To control their concentration and avoid pollution, European directives have defined threshold concentrations for each highly toxic metal. Among them, cadmium, mercury, nickel and lead are listed as priority substances, and their respective mean annual concentrations in water must not exceed 0.2, 0.0, 8.6 and 1.3 μg/L to ensure the protection of ecosystems and health^3^. Conventional remediation techniques are mainly devoted to marine sediments since they accumulate contaminants. Sediments are dredged and confined with an *in situ* capsule or unloaded on the open seas^4^. However, contaminants present at low concentration (<100 mg/L) and dissolved in the seawater column are not taken into account using these conventional processes^5,6^.

To overcome this lack, new technologies are however developed in sediments and in seawater. For example, in seawater, dissolved metallic contaminant could be precipitated or separated with chemical or electrochemical processes^7,8^. Aerogel materials have a high capacity for ab- and/or adsorption towards dissolved metal target compound and could also be used to remediate dissolved contaminants^9^. Also, technologies based on natural behaviours, called bioremediation, have a strong interest for metallic contaminants remediation in seawater with the use of microorganisms^6^, microalgae^10^ or starfish^11^. Nevertheless, all these techniques are very expensive and difficult to implement *in situ*.

Another strategy, economical and using materials easily reachable, could be to control dissolved metals concentrations in seawater by trapping dissolved metallic contaminant in a calcareous deposit. A previous study, tested with nickel ions revealed that it is a promising and cheap clean up device. The principle is to form a calcareous deposit by cathodic polarization of a metallic structure and nickel is removed from seawater by precipitation as nickel hydroxide^12^. This electrochemical technique is mainly used to prevent corrosion of metallic structures in marine environment. It consists in lowering the potential of the metallic structure into its protection domain by application of a fixed current density. This current induces dissolved oxygen and water reductions generating hydroxyl ions production leading to a pH increase at the working electrode interface. The consequence is the co-precipitation of magnesium and calcium naturally present in seawater, as magnesium hydroxide and calcium carbonate as aragonite^13,14^, forming the calcareous deposit^15^.

It was demonstrated that nickel is incorporated in the calcareous deposit in its hydroxide form Ni(OH)_2_ and that this technique seems effective since up to 24% of the nickel initially present in seawater was trapped inside the deposit after only 7 days^12^. So, metallic contaminants trapping is possible when they are expected to form hydroxides.

The aim of the present study is therefore to focus on metallic contaminants trapping as carbonates. Lead is an eligible contaminant for this study for two reasons (i) it is expected to precipitate in its carbonate form, hydrated or not. PbCO_3_ and Pb_3_(CO_3_)_2_(OH)_2_ were expected to form thermodynamic stable species in pH range of cathodic polarization (pH around 9 for oxygen reduction domain and up to 11 for water reduction domain^16–19^; (ii) it is on the list of priority substances from European directives. It has a high toxicity even in small amounts^20^ and although it is very limited and controlled in its modern use, it is still present in environment due to its high utilization during XVIII^e^ and XIX^e^ centuries^21^. The typical concentration of dissolved Pb in seawater is between 2 and 200 ng/L^22–26^ but evidence of marine pollution by lead have been noticed with concentrations up to 14 μg/L^3^.

After studying the behaviour of calcareous deposit in the presence of nickel or lead in the seawater separately, the simultaneous incorporation of the two elements nickel and lead in seawater in laboratory was studied. Finally, experiments were conducted on an *in situ* laboratory, installed in a highly anthropogenic maritime area (strong industrial and port activity) of Nouméa, New Caledonia. The chosen zone, likely to contain different metallic elements, makes it possible to test, in real environmental conditions, the ability of the process to trap the metallic elements.

## Results

### Influence of lead addition on the deposit formation

**Figure 1:**
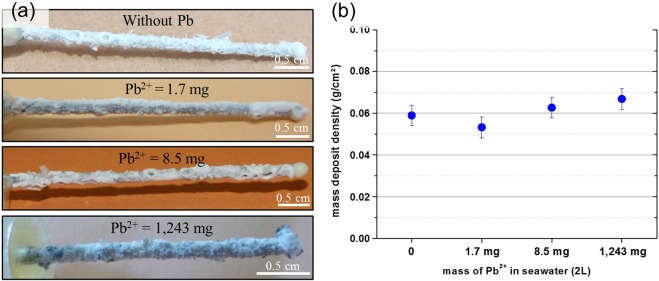
Deposit obtained after 7 days of polarization at −200 μA/cm^2^ with or without PbCl_2_ in seawater. (**a**) Macroscopic pictures, (**b**) mass densities of the deposit. Thickness and mass of the deposit are the same whatever the lead content added in seawater.

Electrochemical monitoring gives information on the chemical reactions that take place at the working electrode interface. At −200 μA/cm², the evolutions with time of the potential were similar with and without lead (see Supplementary Fig. S1). A decrease of the potential response was observed during the first day of polarization. Then, it stabilized at about −1.65 V/SCE after 7 days. Macroscopic pictures of the deposit formed at the galvanized steel wire after 7 days of polarization at −200 μA/cm² with or without PbCl_2_ addition in seawater indicated that the presence of Pb in seawater did not inhibit the deposit formation. The thickness and the mass density of the deposit (normalized by the electrode surface) were always the same whatever the lead content added in seawater (Fig. 1).

### Pb quantification in the deposit

**Figure 2:**
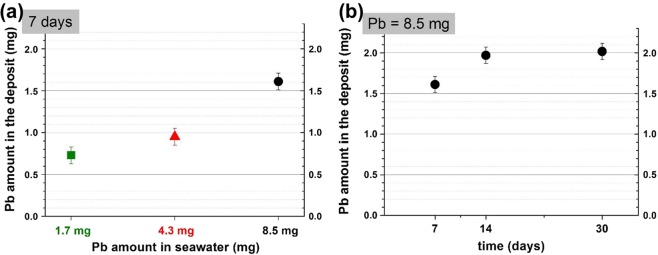
Amount of Pb trapped in the deposit formed at −200 μA/cm² (**a**) as a function of lead content added in seawater (Pb = 1.7 mg; 4.3 mg and 8.5 mg) (**b**) as a function of time with Pb = 8.5 mg (error ± 0.1 mg). The Pb amount inside the deposit increases with the lead content in seawater but stays constant with time after 14 days.

After 7 days of polarization at −200 μA/cm², 0.7, 0.9 and 1.6 mg of Pb trapped in the deposit were measured for respectively 1.7 mg; 4.3 mg and 8.5 mg of initial Pb content in seawater (Fig. 2(a)). During the 7 following days of polarization, Pb content in the deposit continued to slightly increase. It reached 1.97 mg after 14 days for the highest Pb initial concentration (see Fig. 2(b)). This Pb amount remained almost constant after 14 days of polarization. At day 30, a Pb amount of 2.02 mg was quantified inside the deposit and corresponded to about 20% of the total amount.

### Lead characterization in the deposit

**Figure 3:**
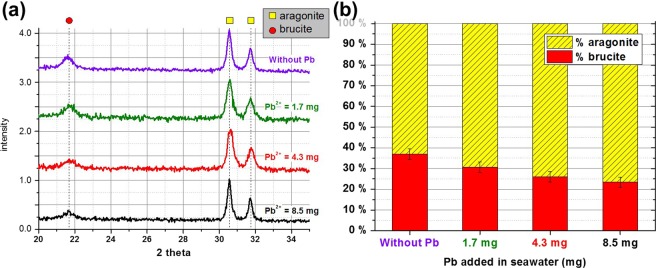
(**a**) X-ray diffraction (XRD) close up spectra for angles 2Ɵ between 18° and 40° obtained showing the presence of CaCO_3_ as aragonite and brucite Mg(OH)_2_^27^. (**b**) Calculated proportions of brucite and aragonite with Rietveld refinement (error ± 10%) for deposits obtained after 7 days of polarization at −200 μA/cm² in seawater without Pb, with Pb = 1.7; 4.3 and 8.5 mg. The brucite phase decreases when the Pb content in seawater increases.

The analysis of the overall deposit by XRD revealed the presence of brucite and aragonite as expected (Fig. 3(a)). Lead compound peaks were not observed. It is probably due to the low concentration of lead compounds in the calcareous deposit therefore they were drowning out in the background of the spectrum. Nevertheless, a decrease of the peak intensity at 2θ = 21.6° attributed to brucite was observed, compare to the CaCO_3_ peaks^27^ with lead concentration increase in seawater. The quantification by Rietveld refinement of the ratio Mg(OH)_2_/CaCO_3_ confirmed this observation (Fig. 3(b)). Indeed, without a Pb added, 37% of the calcareous layer was made of brucite whereas it decreased to 23% with 8.5 mg of lead added in seawater.

**Figure 4:**
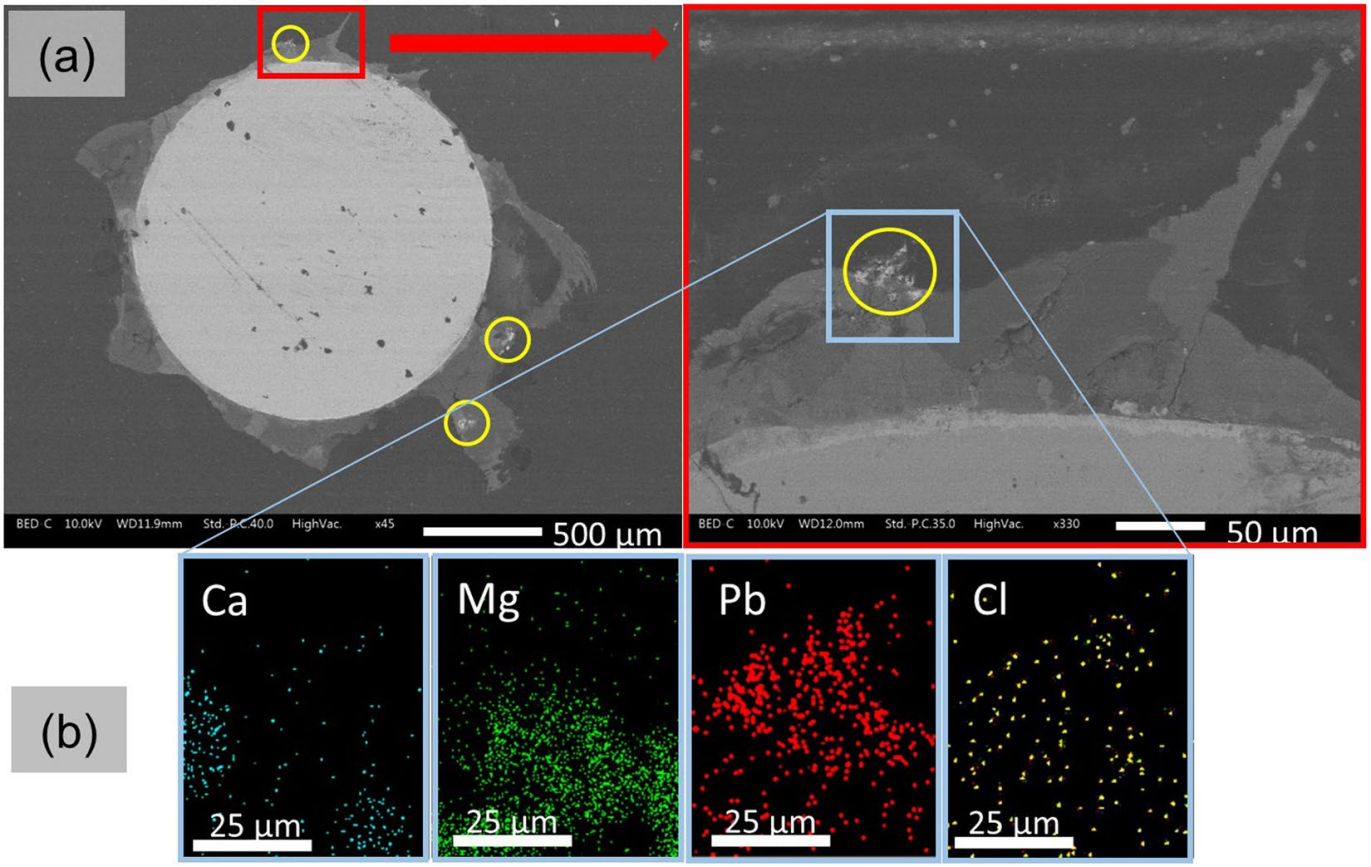
Cross section analysis of the deposit formed after 7 days of polarization at −200 μA/cm² with Pb = 8.5 mg (**a**) SEM picture + enlargement of the red square showing different levels of grey corresponding to different compounds constituting the deposit. (**b**) EDX analysis inside the blue square reveals that the bright grey is composed of lead. The deposit is composed of magnesium (dark grey), calcium (middle grey) and lead (bright grey, yellow surrounding).

A typical cross-section sample image of the electrode after deposit formation during 7 days at −200 μA/cm^2^ was analysed by SEM/EDX and is presented in Fig. 4. The deposit is visible between the steel wire (white central circle) and the resin (external dark background) (Fig. 4(a)). It is mainly composed of dark grey and middle grey corresponding to magnesium and calcium respectively, (see SEM/EDX analysis in Fig. 4(b)). However, bright grey spots corresponding to a heavier element can be observed on the external face of the layer (yellow circle). SEM/EDX analysis of one of these area containing bright grey spots pointed out that the deposit contained lead (Fig. 4(b)). This result confirms the presence of lead trapped inside the calcareous layer although XRD analyses were not able to detect any Pb compounds. However, these Pb spots were located on the outer edge of the deposit and PbCl_2(s)_ particles arising from the seawater embedded in the outer part of the calcareous layer could be suspected. Indeed, Cl was actually detected by EDX (Fig. 4(b)), but Cl signal was less dense and more scattered than Pb. Therefore, Cl signal could not be related to PbCl_2(s)_ embedded in the deposit but results more likely from free chloride ions in seawater that circulates inside the deposit pores. In fact, among the different forms of Pb compounds that could be form in alkaline seawater (alkalinity due to the cathodic polarisation), the most likely phases that could precipitate are the carbonate forms (PbCO_3_ and Pb(CO_3_)_2_(OH)_2_)^17–19^. Chen *et al*. have demonstrated that lead chloride was converted into lead carbonate in presence of carbonates ions at pH around 10^28^. If the lead signal was not related to PbCl_2(s)_ embedded, it means that dissolved lead in seawater co-precipitated with calcium and magnesium during the formation of the deposit. Lead carbonate compounds exhibit a XRD pattern different from aragonite but the very low concentration of Pb compare to Ca and Mg inside the deposit did not permit their identifications by this technique. One can only observe a slight broadening of the aragonite diffraction peaks that suggest the incorporation of Pb substituted to Ca inside the CaCO_3_ phase. As observed by Mwandira *et al*.^18^, it is thus possible that calcium was substituted by lead in the CaCO_3_ compound forming a mixed compound Ca_(1−x)_Pb_x_(CO_3_). It could explain the higher CaCO_3_/Mg(OH)_2_ ratio observed with the lead concentration increase.

### Co-embedding of lead and nickel

**Figure 5:**
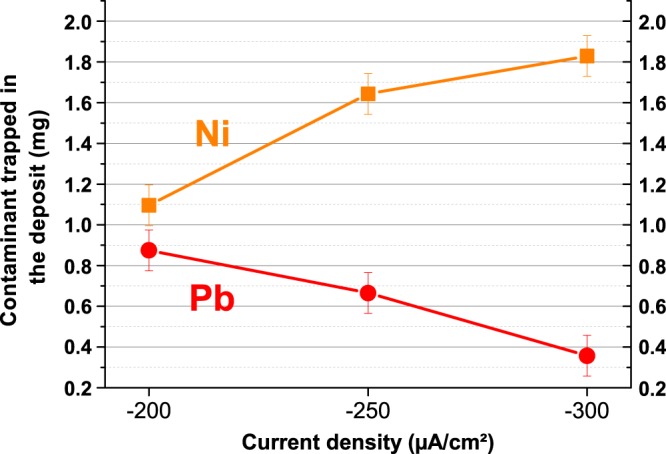
Trapped amount of Pb (red circles) decreases and Ni (orange squares) increases in the deposit as a function of current density, after 7 days of polarization in seawater doped with Ni = 11.7 mg and Pb = 41.4 mg (error ± 0.1 mg).

As nickel could be trapped inside the deposit under its Ni(OH)_2_ form^12^ was previously demonstrated, the possibility to capture both elements Ni and Pb by this process was explored. To do that, experiments with co-addition of nickel and lead in seawater for different applied cathodic current densities (−200 μA/cm^2^, −250 μA/cm^2^ and −300 μA/cm^2^) have been conducted. The quantification of these elements by ICP-OES revealed that the amount of Ni and Pb trapped, as a function of impressed current density, did not exhibit the same tendency (Fig. 5). Nickel trapping was favoured by high current densities and lead trapping was favoured for lower current densities. It is known that hydroxides compounds like brucite are promoted by high current densities^29–31^. In these conditions, the working electrode potential decreases strongly and mainly water reduction takes place, producing a high concentration of OH^−^ ions. It results in a strong increase of the interfacial pH that favours hydroxide forms precipitation. This process consumes the produced OH^−^ and thus inhibits the precipitation of carbonated phases. At a current density of −300 μA/cm², a ratio Mg(OH)_2_/CaCO_3_ of 1.2 was obtained. It appears then consistent that the amount of nickel trapped in the deposit as Ni(OH)_2_ increased with the current density. Lead trapping decreased with the current density. At low current densities, mainly oxygen reduction occurs and OH^−^ ions production is low giving rise to a less alkaline interfacial pH. Carbonate forms like CaCO_3_ or PbCO_3_ are then favoured compared to hydroxide compounds. At a current density of −200 μA/cm², a ratio Mg(OH)_2_/CaCO_3_ of 0.6 was obtained. Lead trapping promoted at low current density confirmed that lead precipitates in carbonate form in the deposit.

### Real conditions experiments

Preliminary *in situ* experiments were performed in an industrial bay of Nouméa, New Caledonia during 7 and 30 days at −300 μA/cm². A white deposit was obtained after experiments (see Supplementary Fig. S2) and it was almost 5x more heavy between 7 and 30 days (Table 1). The cathodic potential, checked periodically, reached −1.65 V/SCE as observed in laboratory experiments. The dissolution and ICP-OES analyses had revealed the presence of Ni and Pb inside the deposit. But at the same time, the presence of Cr and Cu that was already contained in natural seawater was detected^32^. Fe and Zn were also detected but the proportion of each were very high in comparison of the others elements. The working electrode used was composed of steel and zinc as galvanised steel. Even if it was possible that a part came from seawater, it was difficult to distinguish the source of these elements.

**Table 1:** Masses of deposits obtained after 7 and 30 days at −300 μA/cm² in natural seawater and ICP-MS results for Cr, Cu, Fe, Ni, Pb and Zn.

	Mass of deposit obtained (g)	Mass of metallic elements incorporated in the deposit (mg)					
		Cr	Cu	Fe	Ni	Pb	Zn
7 days	14.986	0.3	0.2	25.6	0.6	3.5	360.8
30 days	70.744	1.5	0.5	147.1	3.6	4.7	238.7

## Discussions

Calcareous deposits were formed in presence of lead in seawater, following the same method used in the study of nickel co-precipitation in calcareous deposit^12^. Lead did not modify the calcareous deposit rate growth, even at high concentration in seawater. The potential taken by the electrodes and the deposit features (aspects and mass) were similar for all lead concentration as for that without doped seawater.

The calcareous deposit formation allowed to trap efficiently lead. To have an idea of the efficiency of this method, the ratio (Pb mass trapped)/(initial Pb mass in seawater) was calculate and for respectively 1.7 mg; 4.3 mg and 8.5 mg of initial Pb content in seawater, an average incorporation efficiency of 41%, 21% and 19% in 7 days was obtained. Longer-term experiments, at −200 μA/cm² with 8.5 mg of Pb in seawater, had shown that lead mass in the deposit increased slowly with time. In fact, 19% of the initial lead was trapped in the deposit after 7 days, 23.2% after 14 days and 23.7% after 30 days. Regarding these observations, a limiting factor controlling this process could be mass transport of lead or carbonates. Also, Experiences with −150, −200, −250 and −300 μA/cm² were performed for all Pb concentrations. It was found that at −200 μA/cm², the deposit contained a higher Pb amount than other currents. Lead trapping promoted at low current density confirmed that lead precipitates in carbonate form in the deposit. Assuming that lead precipitates with carbonate ions, CO_3_^2−^ concentration could be a limiting factor for lead precipitation at the interface. At the interface, carbonate ions CO_3_^2−^ production results of bicarbonate ions deprotonation due to hydroxyl ions production and lead is in competition with calcium forming aragonite (CaCO_3_).

This study demonstrated that metallic contaminant could be trapped in the deposit under their hydroxide or carbonate forms. The current density is the driving key parameter for the device since a high current density should be used for contaminant forming hydroxides while a low current density should be used for contaminant forming carbonates. We observed good results for lead and nickel remediation with laboratory experiments for concentrations 1,000x higher than typical concentrations in case of pollution.

In order to confirm these results and to show if other metallic elements naturally present in real seawater could be trapped, *in situ* experiments were performed. Some Cr, Cu, Fe, Ni, Pb and Zn were measured. All masses measured in deposits increased with time. Chromium and nickel masses were 5-fold higher after 30 days. Thermodynamically, these elements were known to precipitate favourably in their hydroxide forms in seawater^33^ (previously confirmed for nickel)^12^. Chromium would therefore have the same behaviour as nickel and would associate with the hydroxyl ions formed by the reductions in water and dioxygen. For copper and lead, the contaminant mass increased by a factor of less than 3 between 7 and 30 days. Copper should preferentially precipitate in its carbonate form within the deposit as the lead^34^. This first *in situ* experiment validated the incorporation in real conditions of contaminants by precipitation with the calcareous deposit. Without seeking an optimal electrochemical condition, it was possible to capture and trap precipitated metallic elements in hydroxide and carbonate form. It is now necessary to perform longer exposure time experiments (6 or 12 months) in laboratory and in real condition with larger metallic fence in order to evaluate the scale effect.

## Methods

### Electrolyte preparation

experiments were performed at room temperature in artificial seawater prepared according to the simplified ASTM D1141 norm^35^ and using the majority of components: NaCl = 24.545 g/L; MgCl_2_, 6H_2_O = 11.101 g/L; Na_2_SO_4_ = 4.091 g/L; CaCl_2_, 2H_2_O = 1.544 g/L; KCl = 0.695 g/L; NaHCO_3_ = 0.235 g/L. 2 L of artificial seawater were used for each experiment, being a large enough volume compared to electrode surfaces (2 cm²) to avoid the depletion of calcium and magnesium ions during the deposit formation.

First, experiments with only Pb doping were performed. Lead chloride salt (PbCl_2_) was added in the artificial seawater after 24 hours of polarization to avoid the metallic lead electrodeposition directly on the electrode^36^. 24 hours are needed to cover the electrode with a thin calcareous deposit layer thus preventing lead deposition as its metallic form. A broad range of lead chloride salt was added in order to have a set of total lead concentration in seawater: 4.1 × 10^−6^; 1.0 × 10^−5^; 2.0 × 10^−5^ and 3.0 × 10^−3^ mol/L *i*.*e*. a mass of total lead of respectively 1.7; 4.3; 8.5 and 1,243 mg in the 2 litres of electrolyte (Table 2). Due to lead solubility in seawater calculated by Visual Minteq Software^37^ the artificial seawater was then saturated and contained a mixture of dissolved (Pb^2+^_(aq)_) and precipitated (PbCl_2(s)_) lead. These high Pb concentrations was chosen in order to validate the process of trapping Pb inside the deposit and at the same time to evaluate a possible modification of the growth rate of the calcareous layer.

**Table 2:** Main characteristics of the laboratory experiments.

Contaminant in seawater		Current density	Experimental time
mol/L	mg	μA/cm²	days
Pb = 4.1 × 10^−6^	Pb = 1.7	−200	7
Pb = 1.0 × 10^−5^	Pb = 4.3	−200	7
Pb = 2.0 × 10^−5^	Pb = 8.5	−200	7; 14; 30
Pb = 3.0 × 10^−3^	Pb = 1,243	−200	7
Pb = Ni = 1 × 10^−4^	Pb = 11.7 + Ni = 41.4	−200	7
Pb = Ni = 1 × 10^−4^	Pb = 11.7 + Ni = 41.4	−250	7
Pb = Ni = 1 × 10^−4^	Pb = 11.7 + Ni = 41.4	−300	7

Secondly, experiments with both nickel and lead doping were performed with a concentration of 1 × 10^−4^ mol//L of Ni and Pb *i*.*e*. a mass of 11.7 mg of Ni and 41.4 mg of Pb in the 2 litres of electrolyte to guarantee no lead nor nickel depletion in the solution for long time experiments (Table 2).

### Electrochemical parameters

**Figure 6:**
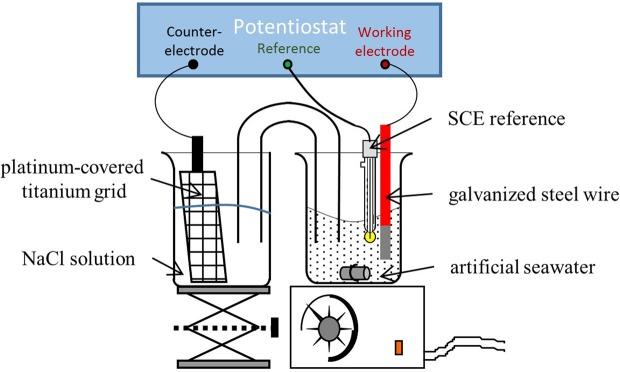
Laboratory experimental setup.

The electrochemical device included a conventional three-electrode cell (Fig. 6) with a commercial galvanized steel wire (l = 5 cm, ∅ = 1.5 mm) as working electrode, a platinum-covered titanium grid as counter electrode and a saturated calomel electrode as reference electrode. Before each experiment, the working electrode surface was degreased with ethanol, cleaned with distilled water and air dried before plunging in seawater. The titanium counter electrode was separated of the working electrolyte by a salty bridge in order to avoid acidification of the electrolytic bath related to the oxidation of the chloride ion to chlorine, acidification of seawater being not compatible with the calcareous deposit formation. This anode was immersed in NaCl solution (30 g/L) and was inert in these conditions (no corrosion). A magnetic stirrer agitated all experiments for renewing solution at the metallic electrode surface. Calcareous deposits were formed by galvanostatic mode (imposed current) for 7, 14 or 30 days. After different tests, a value of −200 μA/cm^2^ was chosen, corresponding to an intermediate current density giving rise to a sufficient calcareous growth rate containing CaCO_3_ in majority. As lead is expected to precipitate under its carbonate form (see discussion), the choice of this current density is then adequate. For co-embedding of lead and nickel, current densities at −200, −250 or −300 μA/cm² for 7 days was applied. The current was produced by a laboratory-made current generator or a potentiostat VSP-BIOLOGIC®. This last equipment was also used to monitor the electrochemical parameters such as potentiometry curves.

### *In situ* experiments

**Figure 7:**
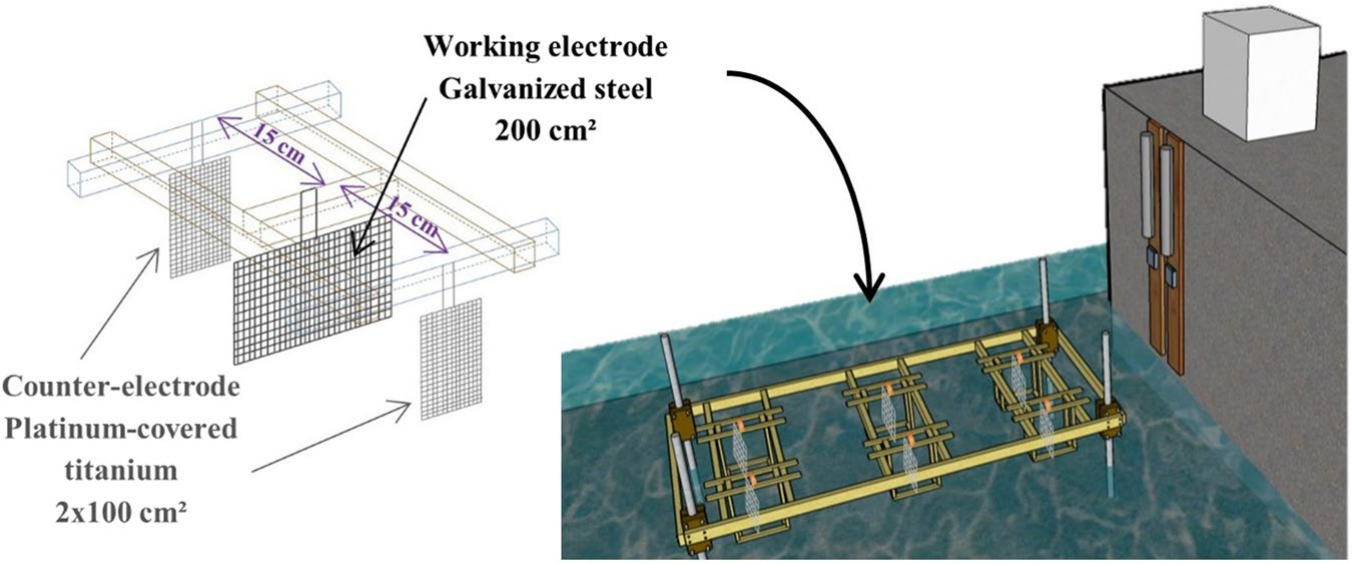
*In-situ* experiments setup.

A preliminary experiment was performed to estimate the device performance in natural conditions. A deported laboratory was installed in Numbo bay, in Nouméa, New Caledonia (22°14′38.3″S, 166°24′56.3″E). This maritime area is strongly entropized (high industrial and harbour activities). Working electrode used was cut out a galvanized steel fence, network of 6 mm (200 cm²). The working electrode was surrounded by two counter-electrodes in platinum titanium to limit the effects of vagabond current (Fig. 7). The calcareous deposit was formed by galvanostatic mode (impressed current) at −300 μA/cm² for 7 and 30 days. Some punctual potential measures were performed manually with a SCE electrode and a multimeter in order to check the potential values and thus the smooth running of the experiments.

### Deposit analysis

At the end of each experiment, electrodes covered with the deposit were rinsed with distilled water in order to remove the excess of dissolved salts. X-Ray Diffraction (XRD) was performed on powdered deposit during 1 h with a RX-Inel with a curved position sensitive detector (CPS-120) using cobalt monochromatic Kα radiation. Rietveld refinements of XRD spectra were performed using Maud software^38^ to determine the aragonite/brucite proportion. Pb distribution inside the deposit was observed on cross section embedded in epoxy resin using Scanning Electron Microscopy coupled with energy dispersive spectrometry (SEM/EDX, JEOL-JSM IT 300) in backscattering mode. Quantifications of Pb trapped in the deposit were performed after deposit dissolution in a nitric acid solution (10%) and measurement by ICP-OES (Varian 730ES Inductively Coupled Plasma-Optical Emission Spectrometry). For deposit obtained *in situ*, considering the large amount of deposit obtained, three replicas of 20 mg were analysed by ICP-MS (Perkin Elmer NexION 350 × Inductively Coupled Plasma-Mass Spectrometry) after dissolution in nitric acid. Six metal elements were analysed: chromium, copper, iron, nickel, lead and zinc. These elements were chosen because, on the one hand, ultramafic Caledonian soils are very rich in iron, nickel and chromium^32^, and on the other hand copper, zinc and lead are metals largely associated with human activities.

## Supplementary information

Supplementary files

## References

[1] Goldhaber SB (2003). Trace element risk assessment: Essentiality vs. toxicity. Regul. Toxicol. Pharmacol..

[2] Kabata-Pendias, A. & Mukherjee B. A. *Trace Elements from Soil to Human*. (Springer, 2007).

[3] The European Parlament and the Council of the European Union. Directive 2013/39/EU as regards priority substances in the field of water policy. Official Journal of the European Union 2013, 1–17 (2013).

[4] Committee on Contaminated Marine Sediments. Contaminated Sediments in Ports and Waterways. (National Academy Press, 1997).

[5] Abdul Jaffar Ali H, Tamilselvi M, Akram AS, Kaleem Arshan ML, Sivakumar V (2015). Comparative study on bioremediation of heavy metals by solitary ascidian, Phallusia nigra, between Thoothukudi and Vizhinjam ports of India. Ecotoxicol. Environ. Saf..

[6] Marques CR (2016). Bio-rescue of marine environments: On the track of microbially-based metal/metalloid remediation. Sci. Total Environ..

[7] Ahalya N, Ramachandra TV, Kanamadi RD (2003). Biosorption of heavy metals. Res. J. Chem. Environmet.

[8] Vasudevan S, Oturan MA (2014). Electrochemistry: As cause and cure in water pollution-an overview. Environ. Chem. Lett..

[9] Maleki H (2016). Recent advances in aerogels for environmental remediation applications: A review. Chem. Eng. J..

[10] Suresh Kumar K, Dahms HU, Won EJ, Lee JS, Shin KH (2015). Microalgae - A promising tool for heavy metal remediation. Ecotoxicol. Environ. Saf..

[11] Hong K-S (2011). Removal of Heavy Metal Ions by using Calcium Carbonate Extracted from Starfish Treated by Protease and Amylase. J. Anal. Sci. Technol..

[12] Carré C (2017). Calcareous electrochemical precipitation, a new method to trap nickel in seawater. Environ. Chem. Lett..

[13] Morse JW, Arvidson RS, Lüttge A (2007). Calcium carbonate formation and dissolution. Chem. Rev..

[14] Devos O (2009). Nucleation-growth process of scale electrodeposition–influence of the magnesium ions. J. Cryst. Growth.

[15] Barchiche C (2003). Characterization of calcareous deposits in artificial seawater by impedance techniques: III. Deposit of CaCO_3_ in the presence of Mg(II). Electrochim. Acta.

[16] Deslouis C, Frateur I, Maurin G, Tribollet B (1997). Interfacial pH measurement during the reduction of dissolved oxygen in a submerged impinging jet cell. J. Appl. Electrochem..

[17] Chen C-TA (1990). Rates of calcium carbonate dissolution and organic carbon decomposition in the North Pacific ocean. J. Oceanogr. Soc. Japan.

[18] Mwandira W, Nakashima K, Kawasaki S (2017). Bioremediation of lead-contaminated mine waste by Pararhodobacter sp. based on the microbially induced calcium carbonate precipitation technique and its effects on strength of coarse and fine grained sand. Ecol. Eng..

[19] Woosley RJ, Millero FJ (2013). Pitzer model for the speciation of lead chloride and carbonate complexes in natural waters. Mar. Chem..

[20] World Health Organisation. Lead poisoning and health. Available at, http://www.who.int/fr/news-room/fact-sheets/detail/lead-poisoning-and-health (Accessed: 29th June 2017) (2016).

[21] French National Assembly and Senate. Rapport sur les effets des métaux lourds sur l′environnement et la santé/Report on the heavy metals effects on the environment and health (2001).

[22] Lin YC, Chang-Chien GP, Chiang PC, Chen WH, Lin YC (2013). Multivariate analysis of heavy metal contaminations in seawater and sediments from a heavily industrialized harbor in Southern Taiwan. Mar. Pollut. Bull..

[23] Paraskevopoulou V (2014). Trace metal variability, background levels and pollution status assessment in line with the water framework and Marine Strategy Framework EU Directives in the waters of a heavily impacted Mediterranean Gulf. Mar. Pollut. Bull..

[24] Martinez-Soto MC (2016). Seasonal variation and sources of dissolved trace metals in Mao Harbour, Minorca Island. Sci. Total Environ..

[25] Zhang J (1995). Geochemistry of trace metals from Chinese river estuary systems - an overview. Estuar. Coast. Shelf Sci..

[26] Munksgaard NC, Parry DL (2001). Trace metals, arsenic and lead isotopes in dissolved and particulate phases of North Australian coastal and estuarine seawater. Mar. Chem..

[27] International Centre for Diffraction Data (ICDD). Powder Diffraction Files (PDF) (1999).

[28] Chen Y, Ye L, Xue H, Yang S (2017). Conversion of lead chloride into lead carbonate in ammonium bicarbonate solution. Hydrometallurgy.

[29] Humble RA (1948). Cathodic protection of steel in seawater with magnesium anodes. Corrosion.

[30] Akamine K, Kashiki I (2002). Corrosion protection of steel by calcareous electrodeposition in seawater Part 1: Mechanism of electrodeposition. Zair. to Kankyo.

[31] Zanibellato, A. Synthèse et études physico-chimiques d’un agglomérat calcomagnésien formé sur acier en milieu marin: un éco-matériau pour la protection du littoral/Synthesis and physico-chemical studies of a calcareous agglomerate formed on steel in marine environment. (Ph.D. Dissertation, La Rochelle, France, 2016).

[32] Gunkel-Grillon P, Laporte-Magoni C, Lemestre M, Bazire N (2014). Toxic chromium release from nickel mining sediments in surface waters, New Caledonia. Environ. Chem. Lett..

[33] Sadiq, M. In *Toxic metal chemistry in marine environments* 154–195 (1992).

[34] Byrne RH, Miller WL (1985). Copper(II) carbonate complexation in seawater. Geochim. Cosmochim. Acta.

[35] *ASTM International*. *Standard for the preparation of substitute ocean water*. *D 1141 - 98* (reapproved 2003) (1998).

[36] Gamburg, Y. D. & Zangari, G. Theory and practice of metal electrodeposition. (Springer Science & Business Media), 10.1007/978-1-4419-9669-5 (2011).

[37] Gustafsson, J. P. Visual Minteq. (2007).

[38] Chateigner, D. Combined analysis. (ISTE, 2010).

