# Supplementary material for: Laboratory and in-situ investigations for trapping Pb and Ni with an unusual electrochemical device, the calcareous deposit in seawater
Source: Sci Rep. 2019 Mar 4;9:3400. doi: 10.1038/s41598-019-40307-0 (PMC6399297; doi:10.1038/s41598-019-40307-0)

Laboratory and in-situ investigations for trapping Pb and Ni with an unusual electrochemical device, the calcareous deposit in seawater

CARRE Charlotte * [a], GUNKEL-GRILLON Peggy [b], SERRES Arnaud [b], JEANNIN Marc [a], SABOT René [a], QUINIOU Thomas [b]

[a] *Laboratoire des Sciences de l'Ingénieur pour l'Environnement LaSIE UMR-CNRS-7356 - Université de La Rochelle, France*

[b] *Institut des Sciences Exactes et Appliquées ISEA EA-7484 - Université de la Nouvelle Calédonie, France*

charlotte.carre@univ-lr.fr

Supplementary Files

Fig. S1 : Evolution with time of the electrode potential with an impressed current density of -200 µA/cm² during 7 days in seawater doped with Pb = 0, Pb = 1.7 mg, Pb = 8.5 mg and Pb = 1243 mg.

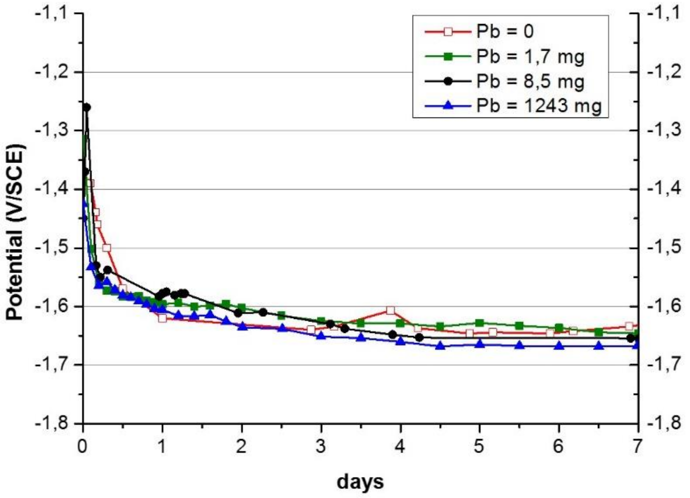

Fig. S2: Deposit obtained after 30 days at -300 µA/cm² in real conditions in an industrial bay of Nouméa, New Caledonia.

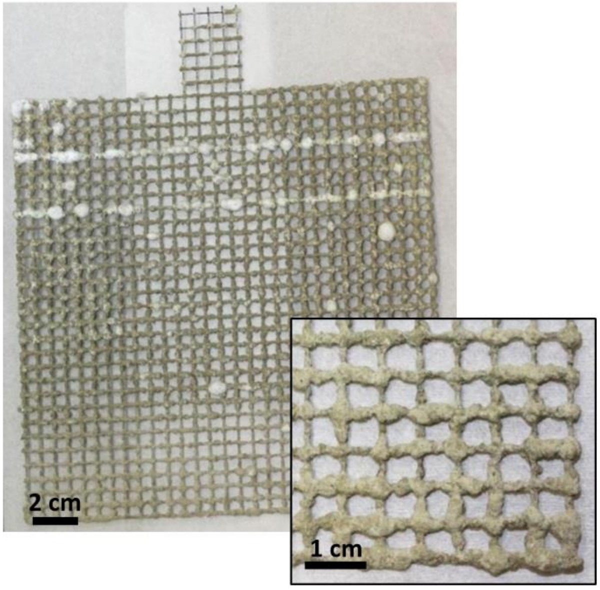

Supplement: Supplementary file 1 — Supplementary files [file 41598_2019_40307_MOESM1_ESM.pdf]
